# Evaluation of bone marrow glucose uptake and adiposity in male rats after diet and exercise interventions

**DOI:** 10.3389/fendo.2024.1422869

**Published:** 2024-06-14

**Authors:** Ronja Ojala, Nicko Widjaja, Jaakko Hentilä, Anna Jalo, Jatta S. Helin, Tuuli A. Nissinen, Niki Jalava, Olli Eskola, Johan Rajander, Eliisa Löyttyniemi, Kaisa K. Ivaska, Jarna C. Hannukainen

**Affiliations:** ^1^ Turku PET Centre, University of Turku, Turku, Finland; ^2^ Institute of Biomedicine, University of Turku, Turku, Finland; ^3^ MediCity Research Laboratory, University of Turku, Turku, Finland; ^4^ Preclinical Imaging Laboratory, Turku PET Centre, University of Turku, Turku, Finland; ^5^ Radiopharmaceutical Chemistry Laboratory, Turku PET Centre, University of Turku, Turku, Finland; ^6^ Turku PET Centre, Accelerator Laboratory, Åbo Akademi University, Turku, Finland; ^7^ Department of Biostatistics, University of Turku, Turku, Finland; ^8^ Department of Biostatistics, Turku University Hospital, Turku, Finland

**Keywords:** bone marrow glucose uptake, bone marrow adiposity, diet, exercise, positron emission tomography

## Abstract

**Objectives:**

Obesity impairs bone marrow (BM) glucose metabolism. Adult BM constitutes mostly of adipocytes that respond to changes in energy metabolism by modulating their morphology and number. Here we evaluated whether diet or exercise intervention could improve the high-fat diet (HFD) associated impairment in BM glucose uptake (BMGU) and whether this associates with the morphology of BM adipocytes (BMAds) in rats.

**Methods:**

Eight-week-old male Sprague-Dawley rats were fed *ad libitum* either HFD or chow diet for 24 weeks. Additionally after 12 weeks, HFD-fed rats switched either to chow diet, voluntary intermittent running exercise, or both for another 12 weeks. BMAd morphology was assessed by perilipin-1 immunofluorescence staining in formalin-fixed paraffin-embedded tibial sections. Insulin-stimulated sternal and humeral BMGU were measured using [^18^F]FDG-PET/CT. Tibial microarchitecture and mineral density were measured with microCT.

**Results:**

HFD rats had significantly higher whole-body fat percentage compared to the chow group (17% vs 13%, respectively; *p* = 0.004) and larger median size of BMAds in the proximal tibia (815 µm^2^ vs 592 µm^2^, respectively; *p* = 0.03) but not in the distal tibia. Switch to chow diet combined with running exercise normalized whole-body fat percentage (*p* < 0.001) but not the BMAd size. At 32 weeks of age, there was no significant difference in insulin-stimulated BMGU between the study groups. However, BMGU was significantly higher in sternum compared to humerus (*p* < 0.001) and higher in 8-week-old compared to 32-week-old rats (*p* < 0.001). BMAd size in proximal tibia correlated positively with whole-body fat percentage (r = 0.48, *p* = 0.005) and negatively with humeral BMGU (r = -0.63, *p* = 0.02). HFD significantly reduced trabecular number (*p* < 0.001) compared to the chow group. Switch to chow diet reversed this as the trabecular number was significantly higher (*p* = 0.008) than in the HFD group.

**Conclusion:**

In this study we showed that insulin-stimulated BMGU is age- and site-dependent. BMGU was not affected by the study interventions. HFD increased whole-body fat percentage and the size of BMAds in proximal tibia. Switching from HFD to a chow diet and running exercise improved glucose homeostasis and normalized the HFD-induced increase in body fat but not the hypertrophy of BMAds.

## 1 Introduction

Bone marrow adipose tissue (BMAT), found mostly in the medullary canal of long bones, constitutes roughly 10% of total adipose tissue mass in humans (1) and comprises of adipocytes (BMAds) that are morphologically similar to white adipocytes (1, 2). However, studies suggest that BMAT is an adipose depot that is distinct from extramedullary adipose tissue (1–4). BMAT responds to changes in energy metabolism by modifying the size and amount of BMAds. In tibia, the basal characteristic of BMAds revealed two anatomically-distinct subpopulations in mice: loosely-packed small BMAds in the proximal tibia and a dense network of large BMAds in the distal tibia referred to herein as regulated and constitutive BMAds, respectively (5–7). Physiologically, constitutive BMAds are less responsive than regulated BMAds to external stimuli such as β-adrenergic stimulation (4) and caloric restriction (8), and are associated with cortical bone loss (9). Regardless, the general expansion of BMAds during both caloric surplus and deficit have been shown to affect local bone metabolism (10–12) and also global energy metabolism (1, 13). Metabolically, adult bone marrow (BM), comprising mostly of BMAT, has been shown to have high basal glucose uptake in humans, exceeding that of white adipose tissue (3, 13). This suggests that BMAT may play a role in whole-body glucose metabolism.

We have previously shown that BMAT-enriched femoral BM insulin-stimulated glucose uptake (GU) and fasting free fatty acid uptake are impaired in insulin resistance in humans (14). Further, lumbar vertebral BM metabolism is affected by body weight (15). We also showed that BMGU is affected by exercise training and the effects of short- and long-term exercise training on BMGU varied according to anatomical location (14, 15). Femoral insulin-stimulated BMGU was increased already after two weeks of cycling exercise and associated positively with whole-body insulin sensitivity and bone turnover rate at baseline. On the contrary, lumbar vertebral BMGU remained unaffected by short-term exercise training, but was higher in participants with high body weight while six months of regular exercise training decreased lumbar vertebral BMGU to the level of leaner participants even without reduction in body weight or BM adiposity (15).

In our previous studies in humans, we used positron emission tomography (PET) to assess the changes in BM metabolism (3, 14, 15). This imaging modality allowed us to study the metabolism of the BM niche as a whole. However, we could not differentiate between the different tissues of BM, including BMAT, trabecular bone and hematopoietic cells, or study the mechanisms underlying these changes. In this study, to gain insight into the effects and mechanisms of weight gain, insulin resistance, and exercise training on BM in more detail, we used PET/CT to evaluate humeral and sternal BMGU and histological analyses to assess tibial BM adiposity. Rats were fed either chow or high-fat diet (HFD) for 24 weeks. Additionally, after 12 weeks, HFD-fed rats switched either to chow diet, voluntary running exercise, or both for another 12 weeks. We hypothesize that HFD impairs insulin-stimulated BMGU and increases BM adiposity, and that these findings can be ameliorated by switch to chow diet and/or running exercise.

## 2 Methods

### 2.1 Animals

This study is part of a larger study entitled CROSRAT with detailed protocols described previously (16). The study was approved by the State Provincial Office of Southern Finland (permission number ESAVI/4080/2019). Male Sprague-Dawley rats were housed in standard cages of two to four rats in an environmentally controlled facility (12/12-h light-dark cycle, 21°C, 55% humidity) at the Central Animal Laboratory of the University of Turku (Turku, Finland). Food and water were provided *ad libitum*.

### 2.2 Diet and exercise interventions

**Figure 1:**
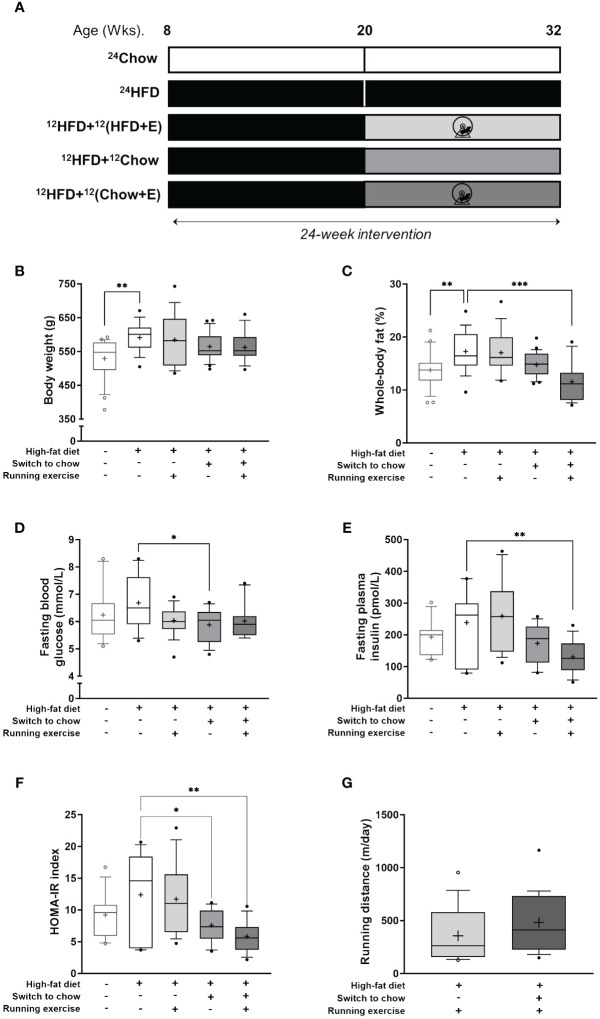
Anthropometrics. **(A)** Study outline. Eight-week-old rats were fed either chow or high-fat diet (HFD) for 24 weeks. Additionally, after 12 weeks, HFD-fed rats switched either to chow diet, voluntary running exercise, or both for another 12 weeks. **(B)** Body weight and **(C)** whole-body fat-percentage at the end of study (*n* = 16–24/group). Fasting plasma levels of **(D)** glucose (*n* = 12–18/group) and **(E)** insulin (*n* = 11–16/group). **(F)** Homeostatic model assessment for insulin resistance (HOMA-IR index; *n* = 11–16/group). **(G)** Mean daily running distance of exercising rats (^12^HFD+^12^(HFD+E), *n* = 16; and ^12^HFD+(^12^Chow+E), *n* = 19). Data is presented as boxes representing quartiles and whiskers representing the 10^th^ and 90^th^ percentiles. Median is marked with a single line and mean value is marked with a plus sign in each box. One-way ANOVA with Dunnet’s correction for multiple comparison was performed in **(B–F)** to compare mean values between groups. The ^24^HFD group served as the control group. Student’s t-test was performed to compare mean difference between groups in **(G)** * *p* < 0.05, ** *p* < 0.01, *** *p* < 0.001.

The study design is shown in **Figure 1A**. Eight-week-old rats were divided into five intervention groups (*n* = 16–24/group) for 24 weeks as follows: (I) age-control group fed with chow diet for 24 weeks (^24^Chow); (II) group fed with HFD for 24 weeks (^24^HFD); (III) exercise intervention group fed with HFD for 24 weeks with the possibility of voluntary running exercise for the last 12 weeks of the intervention (^12^HFD+^12^(HFD+E)); (IV) diet intervention group with an initial 12 weeks of HFD then a switch to chow diet for another 12 weeks (^12^HFD+^12^Chow); and (V) combined intervention group with an initial 12 weeks of HFD then a switch to chow diet with a possibility of voluntary running exercise for the last 12 weeks of the intervention (^12^HFD+^12^(Chow+E)). Additionally, eight-week-old rats without intervention (baseline, age 8 wks, ^0^Chow, *n* = 9) and fed with chow diet for 12 weeks (^12^Chow, age 20 wks, *n* = 6) were imaged with PET/CT to elucidate the effects of aging.

The rats were fed *ad libitum* either standard chow (RM3 (E) Soya free; Special Diets Services, Shropshire, United Kingdom; 15.43 MJ/kg: 11.5% from fat, 27.0% from protein, 61.5% from carbohydrates), or HFD (Western diet, 1.5% cholesterol; ssniff Spezialdiäten GmbH, Soest, Germany; 21.8 MJ/kg: 42.0% from fat, 15.0% from protein, 43.0% from carbohydrates) for 12 or 24 weeks according to the study design (16).

For the exercise groups, rats were housed individually in cages with free access to running wheels (Intellibio, Seichamps, France) from 4 pm to 8 am for four consecutive days a week followed by three days of rest. Individual running distance was recorded for each running day with the activity wheel system and ActiviWheel v.4.4 software (Intellibio, Seichamps, France).

### 2.3 Body composition measurement and blood samples

After the intervention, animals were weighed and body composition was measured with EchoMRI™ 1100 Analyzer (EchoMRI LLC; Houston, TX, USA). Four days before the imaging studies, fasting (4-h) blood glucose was measured from the lateral tail vein with a glucometer (Contour XT; Bayer, Leverkusen, Germany). Blood samples were collected into lithium-heparin plasma collection tubes, and plasma was stored at -80°C. Fasting plasma insulin was measured according to the manufacturer protocol (Mercodia, Uppsala, Sweden). HOMA-IR index was calculated using fasting blood glucose and insulin values (fasting glucose (mmol/l) × fasting insulin (µU/ml)/22.5).

### 2.4 [^18^F]FDG-PET imaging during euglycemic-hyperinsulinemic clamp

At the end of the intervention, rats (*n* = 8–13/group) were fasted (4-h), anesthetized, and prepared for [^18^F]FDG-PET/CT (Inveon Multimodality PET/CT device; Siemens Medical Solutions, Knoxville, TN, USA) during euglycemic-hyperinsulinemic clamp as previously described in detail (16). Insulin clamp was initiated with a 120 mU/kg/min infusion for 3 minutes, followed by 60 mU/kg/min infusion until the blood glucose level dropped to 6.0 mmol/L. Then the insulin infusion was lowered to 18 mU/kg/min for the rest of the clamp protocol. Glucose infusion (20% v/v) was started at the same time. Arterial blood glucose was measured (Contour XT, Bayer, Leverkusen, Germany) before the initiation of the clamp, every 3 minutes for the first 25 minutes and every 5 minutes onwards. After a steady blood glucose level of approximately 5 mmol/L was reached, a 45-min PET scan was started simultaneously with an intravenous injection of 20.7 MBq (SD 1.1) [^18^F]FDG (16).

Whole-body insulin sensitivity (M-value) was calculated from at least a 20-minute time-period with a steady blood glucose level (~5 mmol/L) as previously described (17). After the scan, rats were sacrificed under terminal anesthesia. Subsequently, tibias (*n* = 4–10/group) were collected, fixed in 10% neutral-buffered formalin, and stored in 70% ethanol until further analysis.

### 2.5 Insulin-stimulated BM glucose uptake measurement

**Figure 2:**
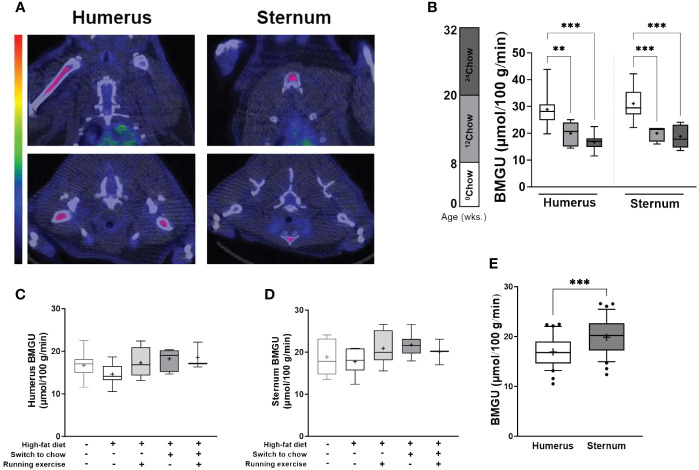
BM insulin-stimulated GU is site- and age-dependent. **(A)** An example of the regions of interest in coronal (top panel) and axial (bottom panel) planes (highlighted in pink) for the analyses of insulin-stimulated bone marrow glucose uptake (BMGU) in humeral and sternal bone marrow from PET/CT images. CT scans were used as anatomical reference. Color bar represents increasing concentration of radioactivity (higher in red). **(B)** Sternal and humeral BMGU are higher at 8 weeks of age than those of older animals in the control chow group (*n* = 6–9/group). There is no difference at 32 weeks in **(C)** humeral or **(D)** sternal BMGU between the intervention groups (*n* = 3–8/group). **(E)** Sternal BMGU is significantly higher than humeral BMGU at 32 weeks when all intervention groups are combined (*n* = 32). Data is presented as boxes representing quartiles and whiskers representing the 10^th^ and 90^th^ percentiles. Median is marked with a single line and mean value is marked with a plus sign in each box. Covariance analysis with Tukey’s correction for multiple comparison was performed in **(B)** and one-way ANOVA with Dunnett’s correction for multiple comparison was performed in **(C, D)** to compare mean values between groups. The ^24^HFD group served as the control group for Dunnett’s comparison. Student’s t-test was performed to compare mean difference between groups in **(E)** * *p* < 0.05, ** *p* < 0.01, *** *p* < 0.001.

For the analysis of BMGU, animals with higher than intended insulin infusion rate or those with incomplete PET-CT image sets were excluded. CT images were acquired as anatomical reference. Insulin-stimulated BMGU was measured from successful PET imaging data (*n* = 3–8 animals/group) using Carimas software (https://carimas.fi) (16). Three-dimensional regions of interest (ROIs) were manually drawn into the BM cavities of the humeri (mid-diaphysis) and the sternum (**Figure 2A**). BM time-activity curves (TACs) were computed from the dynamic PET images for each ROI (16). Due to extensive noise in the measured blood TACs, a multiexponential function was fitted to the measured blood TAC to remove the noise (16, 18). Fractional uptake rate (FUR) was calculated as a ratio of average BM radioactivity concentration between 30 and 45 min after tracer injection and integral of blood TAC. FUR approximates the net influx rate (Ki) (19), and metabolic rate of glucose in BM was estimated by multiplying FUR by glucose concentration in the blood (20).

### 2.6 Quantitation and morphological analysis of BM adipocytes

The histological analysis of BM adiposity in the tibia has been previously described in detail (7). Briefly, tibias (*n* = 4–10/group) were fixed in 10% neutral-buffered formalin overnight and decalcified in a decalcifying solution (10% EDTA, 1 M NaH_2_PO_4_, 0.5 M Na_2_HPO_4_) for 16 days. Decalcified tibias were oriented longitudinally for paraffin embedding and trimmed approximately 40% deep from the surface and sectioned as 4 µm thick sections at the Histology core facility of the Institute of Biomedicine, University of Turku, Finland.

Histological bone sections were then stained with 2 µg/mL rabbit polyclonal anti-perilipin1 (Thermofisher, Massachusetts, USA; PA1–1051, AB_2167579) and 2 μg/mL Alexa Fluor^®^ 594-conjugated goat polyclonal anti-rabbit IgG (Abcam, Cambridge, UK; ab150080, AB_2650602) to visualize BMAds. The slides were then imaged with Pannoramic Midi Slide scanner (3DHISTECH, Budapest, Hungary). Two 1 mm × 1 mm ROIs were drawn into the tibia, one region around the proximal diaphysis 9 mm distal from the proximal growth plate, and the other region non-specifically around the adipocyte-rich BM region of the distal metaphysis, representing analysis regions from proximal and distal marrow of the tibia, respectively (**Supplementary Figure S1A**). Detected adipocyte objects between 200–4000 µm^2^ were included. BMAd density (N.Ad/mm^2^) and individual area (Ad.Ar) were analyzed using the immunofluorescent-based script (Script 2) as described previously (7) in ImageJ version 1.53c (21).

### 2.7 Bone microarchitecture and mineral density

Tibial length was measured with a caliper. Tibia was scanned with µCT SkyScan 1272 (Bruker, Massachusetts, USA) at 70 kV, 135 µA and 10 µm voxel size. The most distal part of the growth plate in the proximal tibia was chosen as the anatomical reference for the analysis. From this reference point, a 2-mm region (200 slices) was selected as the trabecular bone region by tracing the endosteal lining. A 1-mm region (100 slices), 7.5 mm distal to the growth plate, was selected as the cortical bone region by tracing the periosteal lining excluding the marrow cavity. Bone mineral density was measured by referencing the attenuation coefficient of each region to that of phantom materials with known density. The µCT parameters were reported according to the international guidelines (22).

### 2.8 Statistical analyses

The normal distribution of the data was evaluated visually and tested using Shapiro-Wilk test. Pearson’s correlation analysis was performed to determine the correlation between two continuous variables. Mean differences among the groups were analyzed with one-way ANOVA with Dunnett’s correction for multiple comparison. To test the effect of interventions, group (II) served as the high-fat control group (^24^HFD). To test the effect of anatomical BM depot and age, or the difference in BMAd size distribution, covariance analysis with Tukey’s or Dunnett’s correction for multiple comparisons was performed. Difference in running distance between groups was tested using Student’s t-test. Statistical tests were performed in Prism 10 (GraphPad, San Diego, USA) with sample size for each comparison detailed in each figure legend. *P*-values of less than 0.05 (two-tailed) were considered statistically significant.

## 3 Results

### 3.1 Anthropometrics

High-fat feeding (^24^HFD) significantly increased body weight (11.6% higher, *p* = 0.002) and whole-body fat percentage (25.6% higher, *p* = 0.004) when compared to the age-matched chow-fed control group (^24^Chow; **Figures 1B, C**). On average, diet (^12^HFD+^12^Chow), exercise (^12^HFD+^12^(HFD+E)), and combined intervention (^12^HFD+^12^(Chow+E)) decreased body weight and whole-body fat percentage compared to ^24^HFD group. Rats with combined intervention had on average the lowest whole-body fat percentage (11.5%) despite being heavier in body weight than the ^24^Chow group. Further, HFD (^24^HFD) increased the average levels of fasting blood glucose and plasma insulin compared to the ^24^Chow group (**Figures 1D, E**). Dietary intervention alone (^12^HFD+^12^Chow) significantly reduced fasting blood glucose (*p* = 0.03) compared to the ^24^HFD. However, dietary intervention with exercise (^12^HFD+^12^(HFD+E)) had the most pronounced effect in reducing plasma insulin (*p* = 0.005). Whole-body fat percentage correlated positively with weight (r = 0.48, *p* < 0.001), fasting insulin (r = 0.66, *p* < 0.001) and HOMA-IR index (r = 0.64, *p* < 0.001). On average, ^24^HFD group had the highest HOMA-IR index and both diet (^12^HFD+^12^Chow) and combined groups (^12^HFD+^12^(Chow+E)) had significantly reduced HOMA-IR index (*p* = 0.03 and *p* = 0.001, respectively; **Figure 1F**).Whole-body insulin sensitivity (M-value) did not significantly differ between the intervention groups (data not shown). The running distance (m/day) during the intervention was similar in both exercising groups (^12^HFD+^12^(HFD+E)) and (^12^HFD+^12^(Chow+E)) (*p* = 0.16; **Figure 1G**). Running distance correlated negatively with weight (r = -0.47, *p* = 0.005) and whole-body fat percentage (r = -0.52, *p* = 0.002).

HFD (^24^HFD) significantly increased tibial length when compared to the ^24^Chow group (*p* = 0.002; **Table 1**). Further, HFD (^24^HFD) altered trabecular bone microarchitecture in that both trabecular thickness (Tb.Th) and separation (Tb.Sp) were significantly increased (*p* = 0.02 and *p* = 0.002, respectively) and trabecular number (Tb.N) and connectivity density (Conn.D) were significantly decreased (both *p* < 0.001) compared to the ^24^Chow group. Diet intervention (^12^HFD+^12^Chow) significantly increased both trabecular number (*p* = 0.008) and connectivity density (*p* = 0.008) when compared to the ^24^HFD group in that both parameters became comparable to the ^24^Chow group. Group with both interventions (^12^HFD+^12^(Chow+E)) had significantly lower cortical thickness (Cr.Th) compared to the ^24^HFD group (*p* = 0.04). HFD significantly increased endosteal perimeter (Es.Pm; *p* = 0.05). There were no significant differences in bone mineral density (BMD) in trabecular or cortical regions among the groups.

**Table 1:** Tibial length and BMD, trabecular, and cortical parameters obtained by µCT.

Parameter [unit]	^24^Chow	^24^HFD	^12^HFD+ ^12^(HFD+E)	^12^HFD+ ^12^(Chow)	^12^HFD+ ^12^(Chow+E)
*n*	10	6	13	8	4
Bone length [mm]	**42.5** (0.98) **	44.5 (0.42)	43.6 (1.38)	44.2 (0.59)	43.6 (0.08)
Tb.BV/TV [%]	19.4 (2.6)	16.7 (2.2)	17.2 (2.4)	18.9 (1.9)	17.9 (2.7)
Tb.N [1/mm]	**2.64** (0.28) ***	2.05 (0.11)	2.06 (0.19)	**2.47** (0.29) **	2.32 (0.35)
Tb.Th [mm]	**0.073** (0.003) *	0.081 (0.008)	0.083 (0.007)	0.077 (0.003)	0.077 (0.002)
Tb.Sp [mm]	**0.248** (0.021) **	0.309 (0.026)	0.325 (0.023)	0.271 (0.051)	0.292 (0.033)
Conn.D [1/mm^3^]	**180.4** (29.4) ***	108.8 (14.7)	112.7 (13.1)	**163.2** (31.4) **	133.3 (76.9)
Tb.BMD [g/cm^3^]	1.14 (0.02)	1.14 (0.02)	1.17 (0.04)	1.19 (0.06)	1.19 (0.05)
Ct.Th [mm]	0.41 (0.09)	0.49 (0.05)	0.48 (0.06)	0.48 (0.07)	0.36 (0.13)
Ct.Ar [mm^2^]	6.48 (0.89)	7.29 (0.41)	7.10 (0.67)	7.07 (0.58)	7.12 (0.65)
Ct.Ar/Tt.Ar [%]	96.1 (0.8)	95.7 (1.0)	95.7 (0.9)	96.0 (1.3)	94.7 (1.7)
Ps.Pm [mm]	30.30 (3.82)	31.25 (0.74)	31.15 (2.86)	30.65 (2.10)	33.29 (4.58)
Es.Pm [mm]	**27.67** (1.65) *	29.56 (0.85)	28.53 (1.47)	28.69 (1.48)	28.32 (1.23)
Ct.BMD [g/cm^3^]	1.25 (0.01)	1.26 (0.00)	1.25 (0.02)	1.27 (0.02)	1.28 (0.04)

Tb.BV/TV, trabecular bone volume per tissue volume; Tb.N, trabecular number; Tb.Th, trabecular thickness; Tb.Sp, trabecular thickness; Conn.D, connectivity density; Tb.BMD, trabecular bone mineral density; Ct.Th, cortical thickness; Ct.Ar, cortical area; Ct.Ar/Tt.Ar, cortical area per tissue area; Ps.Pm, periosteal perimeter; Es.Pm, endosteal perimeter; Ct.BMD, cortical bone mineral density. Data is presented as mean (SD). Statistical differences analyzed with one-way ANOVA using Dunnet’s adjustment for multiple comparison and ^24^HFD as reference. Bold numerals represent statistically significant comparison. Adjusted p-values are presented * p < 0.05, ** p < 0.01, *** p < 0.001.

### 3.2 BMGU is age and site-dependent but is not significantly affected by the intervention

The level of BMGU was age and site-dependent in the chow group. Sternal and humeral BMGU being significantly higher in 8-week-old (^0^Chow) than in 20-week-old (^12^Chow; *p* < 0.001 and *p* = 0.005, respectively) and 32-week-old rats (^24^Chow; both *p* < 0.001) (**Figure 2B**). At 32 weeks, there was no statistically significant difference in BMGU between any of the intervention groups (**Figures 2C, D**). However, despite humeral BMGU was similar among the intervention groups (*p* = 0.21), it tended to decrease in ^24^HFD when compared to ^24^Chow and was normalized by diet and exercise interventions (**Figure 2C**). Sternal BMGU was significantly higher than humeral BMGU when all animals were included in the analysis at 32 weeks (*p* < 0.001) (**Figure 2E**).

### 3.3 Exposure to high-fat diet increases BM adiposity which is not reversed by diet or exercise intervention

**Figure 3:**
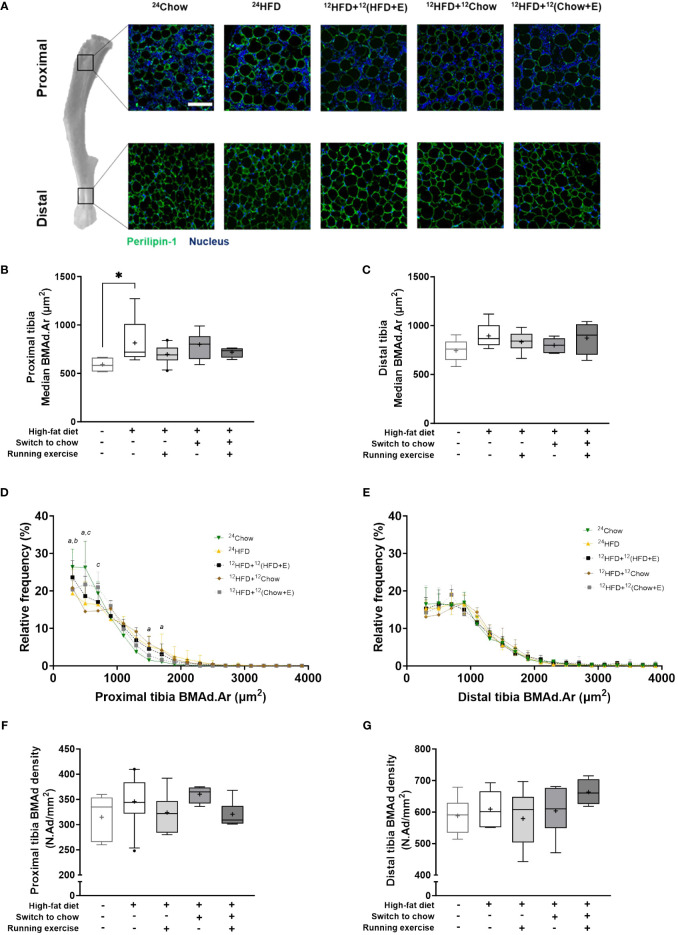
BMAds resist diet-induced hypertrophy in response to a dietary and/or exercise intervention. **(A)** Differences in the morphology of bone marrow adipocytes (BMAds) in different regions of the tibia. BMAds in the proximal tibia are smaller and more interspersed with bone marrow cells than those in the distal tibia. Scale bar represents 100 µm. High-fat diet increases the median size of BMAds in **(B)** the proximal tibia, but not in **(C)** the distal tibia. Frequency distribution of the size of BMAds in **(D)** proximal and **(E)** distal tibia. A bin width of 200 µm^2^ was applied from a lower and upper bin center of 300 µm^2^ and 3900 µm^2^, respectively. Small letters indicate a significant difference in the mean frequency distribution between the high-fat group (^24^HFD) and age-matched group (^24^Chow, *a*) or the exercising group (^12^HFD+^12^(HFD+E), *b*) or the combination group (^12^HFD+^12^(Chow+E), *c*). **(F, G)** The density of BMAds is not affected by study intervention. One-way ANOVA with Dunnett’s correction for multiple comparison was performed in **(B, C, F, G)** to compare mean values between groups. Data is presented as boxes representing quartiles and whiskers representing the 10^th^ and 90^th^ percentiles. Median is marked with a single line and mean value is marked with a plus sign in each box. Covariance analysis with Dunnett’s correction for multiple comparison was performed in **(D, E)** to compare group wise mean frequency distribution in each bin. The ^24^HFD group served as the control group for Dunnett’s comparison. *n* = 4–10/group. * *p* < 0.05.

BM adiposity was evaluated in histological tibial sections (**Figure 3A**). The median size of regulated BMAds in the proximal tibia was significantly larger in ^24^HFD group than in ^24^Chow group (815 µm^2^ versus 592 µm^2^, *p* = 0.03; **Figure 3B**), indicating a hypertrophic response of regulated BMAds with high-fat diet. However, the hypertrophy of constitutive BMAds was not evident in the distal region of the tibia (**Figure 3C**). On average, diet, exercise, and combined intervention slightly decreased the median size of BMAds in the tibia, although this was not statistically significant and the size of BMAds was still larger than in ^24^Chow group at 32 weeks. The median size of constitutive BMAds was significantly larger than that of regulated BMAds (822 µm^2^ versus 716 µm^2^, *p* = 0.003, data not shown) at 32 weeks. The frequency of small regulated BMAds (200–600 µm^2^) in the proximal tibia was lower in the ^24^HFD group compared to the ^24^Chow group (*p* < 0.001). In line with this, the frequency of large regulated BMAds (1200–1600 µm^2^) was higher in the ^24^HFD group compared to the ^24^Chow group (**Figure 3D**). The size distribution of constitutive BMAds was similar across all groups (**Figure 3E**). Histologically, regulated BMAds were more interspersed with BM cells than constitutive BMAds. Regardless, the density of BMAds was similar in both analysis regions in the tibia after interventions (**Figures 3F, G**). The analysis of BM adiposity by area in our selected 1 mm^2^ proximal regions matched that in whole BM section quantitated with MarrowQuant (23) (**Supplementary Figure 1**).

### 3.4 Humeral, but not sternal, BMGU correlates with the size of BMAds in proximal tibia

**Figure 4:**
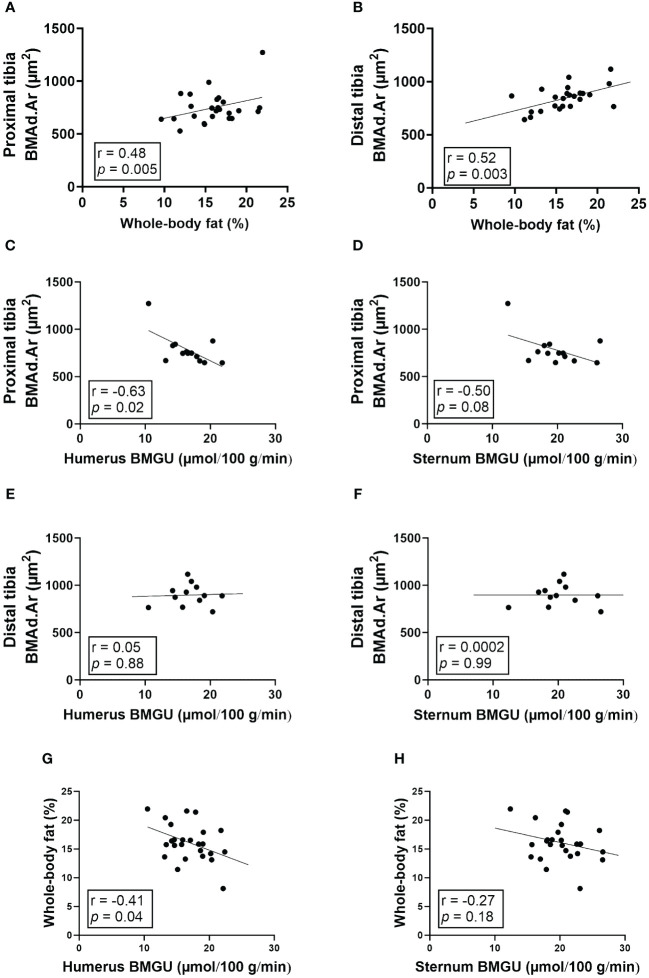
The size of BMAds in the proximal tibia inversely correlates with humeral BMGU. **(A, B)** The size of bone marrow adipocytes (BMAds) in proximal (*n* = 32) and distal (*n* = 31) tibia correlates positively with whole-body fat percentage. In rats exposed to high-fat diet, **(C-F)** the size of BMAds in the proximal tibia (*n* = 16), but not distal (*n* = 15), inversely correlates with humeral, but not sternal, insulin-stimulated bone marrow glucose uptake (BMGU). In addition, **(G, H)** humeral, but not sternal, BMGU correlated with whole-body fat percentage (*n* = 25) among rats exposed to high-fat diet. Pearson’s correlation analysis was performed for all analyses.

The size of BMAds correlated strongly with whole-body fat percentage in both proximal (r = 0.48, *p* = 0.005; **Figure 4A**) and distal tibia (r = 0.52, *p* = 0.003; **Figure 4B**). Amongst study groups that were exposed to HFD (study groups II-V), the size of regulated BMAds in the proximal tibia correlated inversely with humeral (r = -0.63, *p* = 0.02; **Figure 4C**) and sternal BMGU (r = -0.50, *p* = 0.08; **Figure 4D**). However, there was no significant correlation between the size of constitutive BMAds in the distal tibia and humeral or sternal BMGU (**Figures 4E, F**). Humeral, but not sternal, BMGU correlated inversely with whole-body fat percentage (r = -0.41, *p* = 0.04; **Figures 4G, H**) in rats exposed to HFD.

## 4 Discussion

In this study, we examined BMGU using [^18^F]FDG-PET/CT during insulin stimulation and BM adiposity using immunohistochemistry in male Sprague-Dawley rats. Eight-week-old rats were exposed to either chow or HFD for 24 weeks. Additionally after 12 weeks, HFD-fed rats switched either to chow diet, voluntary running exercise, or both for another 12 weeks. A long-term 24-week exposure to HFD markedly increased body weight, whole-body adiposity and induced hypertrophy of regulated BMAds in the proximal tibia, consistent with previous findings in mice (11). The group with double intervention (^12^HFD+^12^(Chow+E)) had the most pronounced reduction in whole-body fat percentage when compared to ^24^HFD and ^24^Chow groups. HOMA-IR index, a measure of insulin resistance, was significantly lower in both groups that switched to chow diet compared to ^24^HFD group which suggests an improved insulin sensitivity. Interestingly, voluntary wheel running during HFD did not affect the whole-body fat percentage when compared to the sedentary ^24^HFD. HFD affected mainly the trabecular region of bone implying trabecular bone loss, which is likely due to higher bone metabolism in that region responding to the intervention, similar to what has been reported elsewhere (6, 24).

BMGU was higher in 8-week-old rats compared to 32-week-old rats in both humeral and sternal BM. This phenomenon may be explained by the function of BM and the physiological “red-to-yellow” transition. The BM cavity of young rodents as well as humans consists predominantly of hematopoietic tissue, which during aging is replaced by adipocyte-rich yellow BM (5, 23, 25). The proliferation and differentiation of hematopoietic stem cells into different blood cell types require a robust upregulation of energy metabolism compared to less metabolically active BMAT in older rodents (26). Also, the need for energy caused by growing may be reflected in the BMGU. Taken together, high BMGU in younger rats may represent more active hematopoiesis and bone formation than low BMGU in skeletally mature and BMAT-enriched marrow of older rats.

At 32 weeks, sternal BMGU was significantly higher than humeral BMGU when all intervention groups were combined. Cline et al. showed in rats that cellular composition of humeral and sternal BM is different as sternal BM contains more hematopoietic tissue (27). Our data agrees with this finding, which is reflected by higher BMGU in the sternum than in humerus. The same phenomenon can be seen in humans when comparing vertebral and femoral BMGU (14, 15, 28). In humans, lumbar vertebral BM is more hematopoietically active while femoral BM cavity serves mainly as a specialized fat depot (14, 15, 28). Additionally, we found that humeral, but not sternal, BMGU with HFD exposure correlated inversely with whole-body fat percentage. It seems that higher BMGU tied to hematopoiesis in the axial skeleton is more independently regulated than BMGU in BM of long bones. Our data suggests that even small differences in BM cellularity can result in significant differences in BM metabolism and GU.

Despite differences in body weight, whole-body fat percentage, and glycemic status we did not observe any statistically significant differences in BMGU among intervention groups in either humerus or sternum. However, on average it seemed that both switching to chow and running exercise (^12^HFD+^12^(Chow+E)) increased BMGU compared to ^24^HFD group. This agrees with our previous findings in humans, where we found that femoral BMGU increased in response to exercise training similarly in both healthy and insulin-resistant participants after two weeks of exercise training (14), and in co-twins discordant for body weight after six months of regular exercise training (15). However, in our previous twin study we showed that exercise training induced an increase in BMGU even without a decrease in BM adiposity measured using CT radiodensity (15). According to our data, it seems that this same phenomenon can be seen in rats and changes in BMGU and adiposity can occur independent of each other. To our knowledge, there are no other studies on BMGU under euglycemic-hyperinsulinemic clamp; however, Suchacki et al. compared the effects of insulin and vehicle on BMGU in fasting condition without continuous glucose infusion (13).

One reason that could explain the similar BMGU and whole-body insulin sensitivity (M-value) among intervention groups, unlike our previous findings in humans, is that rats were anesthetized with isoflurane while humans were awake during imaging (14–16, 28). Isoflurane anesthesia has been shown to cause hyperglycemia in humans and in rats (29–31). The increased levels of plasma glucose are a consequence of impaired insulin secretion, impaired glucose clearance and increased glucose production (29, 31). Indeed, anesthesia is needed to immobilize rodents for imaging, which might have affected the reliability of the parameters related to glucose measurements reported in this study. We have previously shown in humans that BM metabolism can be affected by exercise training (14, 15, 28). However, in this study, no statistically significant changes in BMGU were found in rats. Also, the effect of aging in decreasing insulin-stimulated BMGU may concomitantly mask the effect of our intervention. To elucidate the effect of anesthesia and aging on insulin sensitivity measurements more studies are required.

Although interventions reduced whole-body fat percentage, neither diet nor exercise intervention reduced BM adiposity close to the age-matched control (^24^Chow). Indeed, BMAT has been reported to be distinct from extramedullary adipose depots (1–4), which may explain why BMAT was relatively resistant to the interventions in our experimental model. For instance, the limited lipolytic capacity of BMAds compared to subcutaneous white adipocytes may partly explain the resistance of BMAT to remodel under external stimuli (2, 4). Further, BMAT is presented with higher expression of inflammation-related genes compared to other extramedullary adipose tissues under basal condition (11); this may prime adipogenesis upon early exposure to the HFD. In accordance with this, BM global gene expression showed a pro-adipogenic and pro-inflammatory profile with HFD (32). In their study, rats with exercise intervention, similar to our ^12^HFD+^12^(HFD+E) group, remained pro-adipogenic but not pro-inflammatory when compared to the sedentary HFD rats; this may explain the relatively stable morphology of BMAds observed in our study. However, specific changes in BMAds remain to be investigated.

Interestingly, HFD significantly increased the median size of BMAds in the proximal but not distal tibia, representing diet-specific response in regulated but not constitutive BMAds, similar to the work by Scheller et al. (5). The established heterogeneity may partly explain the regional differences observed in the response of BMAds in the tibia in our model.

Previously, we and others have shown that BMAT-enriched human BM is an insulin-sensitive depot (3, 14, 15, 28). In this study, we show that HFD increased the size of regulated BMAds in the proximal tibia which correlated inversely with insulin-stimulated BMGU in both humerus and sternum, suggesting that BMAT is not protected from obesity. Although more studies are needed to mechanistically link the size of BMAds and BMGU, research on white adipocytes shows the association between adipocyte hypertrophy and insulin resistance (33–35). In this study, the analyzed regions for BM adiposity (proximal and distal tibia) and BMGU (humerus and sternum) were different. The rats were positioned in the PET/CT scanner head first, which means their hind legs were outside the axial field of view of the scanner and thus not included in the analysis. However, BM of the long bones, such as tibia and humerus are considered to be similar in BM cellularity, BMAT volume and BMGU (8, 13, 27).

To the best of our knowledge, this is the first study to combine dynamic [^18^F]FDG-PET imaging during euglycemic-hyperinsulinemic clamp and morphological histology to assess the effects of diet and exercise on BMGU and BM adiposity. While many studies have shown marked reduction in BM adiposity with diet and exercise intervention, our study model showed a rather moderate effect. This may have been the result of the use of less energy-dense (42% calories from fat) diet and voluntary running for four days per week compared to others (6, 36–38). Furthermore, the difference in the duration of the interventions and the age of animals may affect the response of BMAT to the interventions. For instance, aging-related expansion of BMAT (5, 12, 23, 39) may have masked the effect of intervention in older animals. Further, it might be that the effect of the interventions on basic anthropometrics as well as BMAT and BM metabolism could have been more pronounced after a longer or more rigorous exercise intervention or with a diet containing more fat. Our study intervention was designed to reflect the feasibility in clinical practice and provide mechanistic insights that parallels to our previous human studies (14, 15).

In summary, high-fat diet increases whole-body fat percentage and the size of regulated BMAds in the proximal but not that of constitutive BMAds in the distal region of the tibia, suggesting that BMAT is not protected from the effects of obesity. While a 12-week dietary and intermittent running exercise intervention is effective in reducing whole-body fat percentage, it does not significantly affect bone marrow adiposity. The size of regulated BMAds correlate inversely with bone marrow glucose uptake in the humerus. Insulin-stimulated bone marrow glucose uptake is site-specific and decreases with age and the study interventions are not able to affect it. To reverse bone marrow adipocyte hypertrophy and induce favorable changes in bone marrow glucose uptake, a more intense intervention may be required.

## Data availability statement

The raw data supporting the conclusions of this article will be made available by the authors, without undue reservation.

## Ethics statement

The animal study was approved by State Provincial Office of Southern Finland. The study was conducted in accordance with the local legislation and institutional requirements.

## Author contributions

RO: Data curation, Formal analysis, Methodology, Writing – original draft, Writing – review & editing. NW: Data curation, Formal analysis, Investigation, Methodology, Validation, Visualization, Writing – original draft, Writing – review & editing. JH: Writing – review & editing, Data curation, Formal analysis, Methodology. AJ: Writing – review & editing, Investigation. JSH: Writing – review & editing, Conceptualization, Investigation. TN: Writing – review & editing, Investigation. NJ: Writing – review & editing, Formal analysis. OE: Writing – review & editing, Investigation. JR: Writing – review & editing, Investigation. EL: Writing – review & editing, Formal analysis. KKI: Conceptualization, Funding acquisition, Methodology, Project administration, Resources, Supervision, Writing – original draft, Writing – review & editing. JCH: Conceptualization, Funding acquisition, Methodology, Project administration, Resources, Supervision, Writing – original draft, Writing – review & editing.

## Supplementary material

Supplementary Figure 1 Analysis of BM adiposity in our 1 mm x 1 mm ROI matches that in whole BM section. **(A)** Extraction of region of interest (ROI) from the proximal and distal regions of the tibia. Note that the proximal growth plate was cut during sample processing. Scale bars: 3 mm and 100 µm (inserts). **(B)** Bone marrow (BM) adiposity by area analyzed with ImageJ correlates positively with MarrowQuant whole BM adiposity by area (*n* = 33). Groupwise analysis of bone marrow adiposity by area with **(C)** ImageJ from selected ROI or **(D)** MarrowQuant from whole BM section (*n* = 4–10/group). Data is presented as box representing quartiles and whiskers representing 10^th^ and 90^th^ percentiles. One-way ANOVA with Dunnet’s correction for multiple comparison was performed in **(C, D)** to compare mean values between groups. The ^24^HFD group served as the control group for Dunnett’s comparison. * *p* < 0.05.
