# Supplementary figures and images for: Evaluation of bone marrow glucose uptake and adiposity in male rats after diet and exercise interventions
Source: Front Endocrinol (Lausanne). 2024 Jun 14;15:1422869. doi: 10.3389/fendo.2024.1422869 (PMC11211282; doi:10.3389/fendo.2024.1422869)

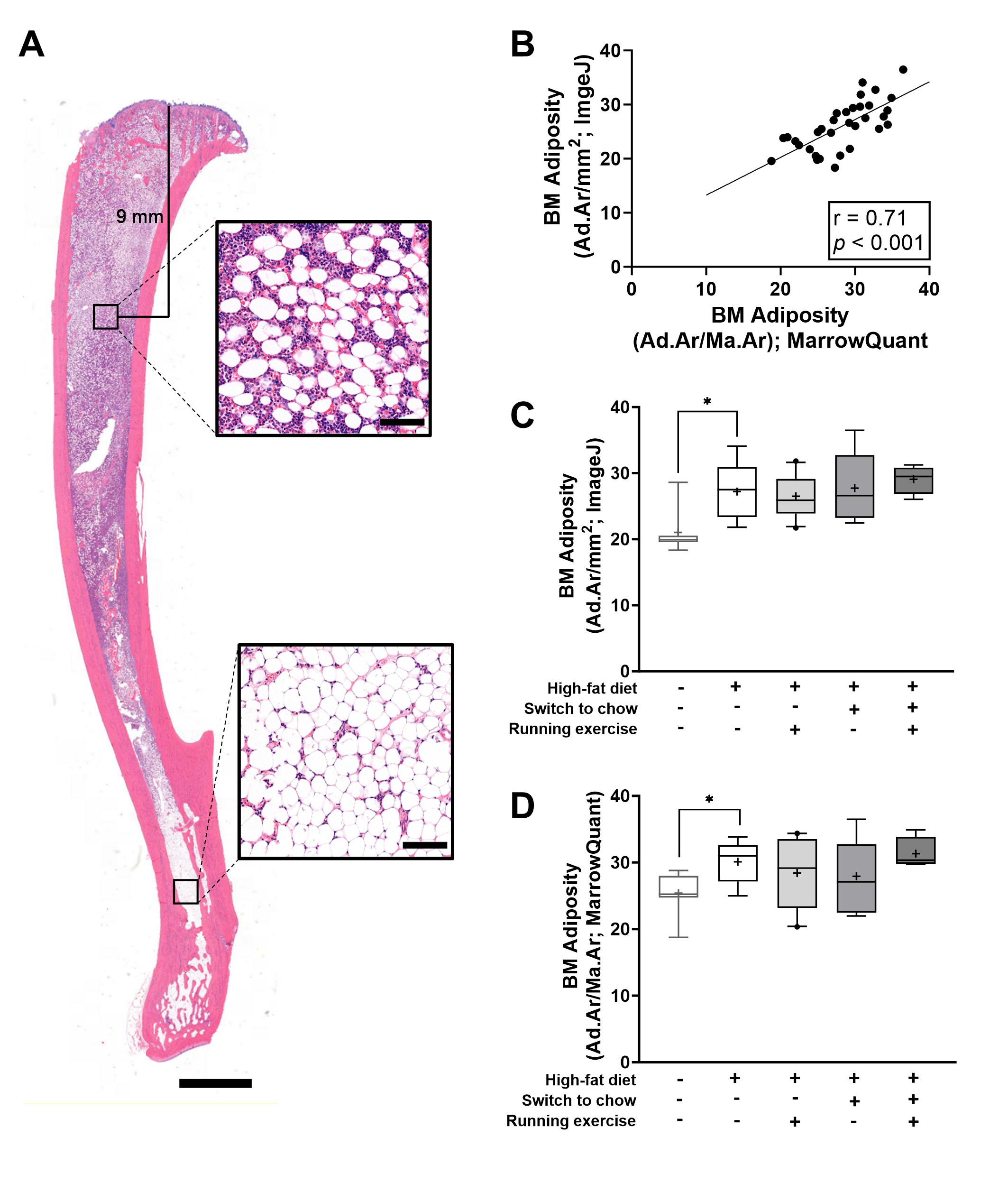

Supplement: Supplementary Figure 1 — Analysis of BM adiposity in our 1 mm x 1 mm ROI matches that in whole BM section. (A) Extraction of region of interest (ROI) from the proximal and distal regions of the tibia. Note that the proximal growth plate was cut during sample processing. Scale bars: 3 mm and 100 µm (inserts). (B) Bone marrow (BM) adiposity by area analyzed with ImageJ correlates positively with MarrowQuant whole BM adiposity by area (n = 33). Groupwise analysis of bone marrow adiposity by area with (C) ImageJ from selected ROI or (D) MarrowQuant from whole BM section (n = 4–10/group). Data is presented as box representing quartiles and whiskers representing 10th and 90th percentiles. One-way ANOVA with Dunnet’s correction for multiple comparison was performed in (C, D) to compare mean values between groups. The 24HFD group served as the control group for Dunnett’s comparison. * p < 0.05. [file Image_1.jpeg]
